# Novel paradigm for immunotherapy of ovarian cancer by engaging prophylactic immunity against hepatitis B virus

**DOI:** 10.1186/s40169-016-0125-2

**Published:** 2016-11-30

**Authors:** Marek Malecki, Emily Putzer, Caroline Quach, Chaitanya Dodivenaka, Xenia Tombokan

**Affiliations:** 1 Phoenix Biomolecular Engineering Foundation, San Francisco, CA USA; 2 University of Wisconsin, Madison, WI USA; 3 National Magnetic Resonance Facility, National Institutes of Health, Madison, WI USA; 4 District of Columbia, Department of Health, Washington, DC USA; 5 Long Island University, Brookville, NY USA; 6 South Dakota State University, Brookings, SD USA; 7 Bruker Bio-Spin Corporation, The Woodlands, TX USA

## Abstract

**Background:**

Only eight women out of one hundred diagnosed with ovarian epithelial cancers, which progressed to the clinical stage IV, survive 10 years. First line therapies: surgery, chemotherapy, and radiation therapy inflict very serious iatrogenic consequences. Passive immunotherapy of ovarian cancers offers only low efficacy. Prophylactic and therapeutic vaccines for ovarian cancers are not available. Interestingly, prophylactic vaccines for Hepatitis B Viruses (HBV) are very effective.

**Specific aim:**

The specific aim of this work was to design, synthesize, and administer biomolecules, which would engage prophylactic, vaccination-induced immunity for HBV towards killing of ovarian cancer cells with high specificity and efficacy.

**Patients:**

Tissue biopsies, ascites, and blood were acquired from the patients, whose identities were entirely concealed in accordance with the Declaration of Helsinki, pursuant to the Institutional Review Board approval, and with the Patients’ informed consent.

**Methods and results:**

By biomolecular engineering, we have created a novel family of biomolecules: antibody × vaccine engineered constructs (AVEC: anti-HER-2 × HBsAg). We have collected the blood from the volunteers, and measured the titers of anti-HBV antibodies resulting from the FDA approved and CDC scheduled HBV vaccinations. We have acquired tumor biopsies, ascites, and blood from patients suffering from the advanced ovarian cancers. We have established cultures of HER-2 over-expressing epithelial ovarian cancers: OV-90, TOC-112D, SKOV-3, as well as human ovary surface epithelial (HOSE) and human artery endothelial (HAE) cells. Treatment of the HER-2+ ovarian cancer cells with AVEC: anti-HER-2 × HBsAg, accompanied by administration of blood drawn from patients with high titers of the anti-HBV antibodies, resulted in much higher therapeutic efficacy as compared to treatment with the naked anti-HER-2 antibodies alone and/or with the relevant isotype antibodies. This treatment had practically no effect upon the HOSE and HAE cells.

**Discussion:**

Herein, we report attaining the great improvement in eradication efficacy of ovarian epithelial cancer cells’ by engaging prophylactic immunity against HBV; thus creating a novel paradigm for immunotherapy of ovarian cancer. We have accomplished that by designing, synthesis, and administration of AVEC. Therefore, the HBV vaccination acquired immunity mounts immune response against the vaccine, but AVEC redirect, accelerate, and amplify this immune response of all the elements of the native and adaptive immune system against ovarian cancer. Our novel paradigm of immunotherapy is currently streamlined to clinical trials also of other cancers, while also engaging prophylactic and acquired immunity.

**Conclusion:**

Novel antibody-vaccine engineered constructs (AVEC) create the solid foundation for redirected, accelerated, and amplified prophylactic, HBV vaccination-induced immunity immunotherapy (RAAVIIT) of ovarian cancers.

## Introduction

### Background

Only eight women out of one hundred diagnosed with ovarian epithelial cancers, which progressed to the clinical stage IV, survive 10 years. More than 70% of all these patients are diagnosed, when the cancer progressed already to this stage IV [1, 2]. Ovarian cancer cells at this stage spread through the peritoneal cavity to other organs. However, the invasive cancer cells are detected in the ascites already from the clinical stage Ic. Progression of this cancer is associated with the changing gene expression profile. It is reflected by expression of the epithelial growth factor receptor 2 (HER-2) reported in up to 30% of all the patients, but in almost all of the patients diagnosed with ovarian cancer cells at the clinical stage IV [3–8].

The first line therapies involve surgery, radiation, and chemo-therapy. Currently recommended first line therapies include oophorectomy, systemic chemotherapeutics with alkylating agents (cisplatin or carboplatin) and M-phase specific tubulin inhibitors (paclitaxel or docetaxel), and radiotherapy (~20 Gy). While saving patients’ lives, these therapies’ cause tremendous iatrogenic side effects, which range from hair loss, through compromised immunity, to permanent infertility. These side effects are far more severe, if the treatments have to include metastases to liver, lungs, or brain. These therapies may also cause secondary cancers resulting from mutagenesis caused by chemo-therapeutics and ionizing radiation. These iatrogenic injuries stimulate research towards personalized, targeted therapeutics including immunotherapy and vaccination.

Clinical trials of immunotherapy employing humanized monoclonal antibodies anti-HER-2: trastuzumab (Herceptin) and pertuzumab (Perjeta), which are very effective in breast and head and neck cancers, result in minimal improvements in treatment of ovarian cancers [9–11]. In essence, immunotherapy, tested in clinical trials, relies upon provision of passive, humoral immunity by intravenous infusion of the humanized mouse monoclonal antibodies. In addition to inhibiting cells’ proliferation by blocking HER-2, these antibodies’ efficacy could rely upon assembling of the patients’ adaptive immune response. However, in patients, exhausted by the disease and rounds of systemic therapy, and with cancer progression over the time needed to assemble that response, it is hardly possible.

Prophylactic and therapeutic (administered after the outbreak of the disease) vaccines for ovarian cancers are not available. For women with high genetic susceptibility of cancer (e.g., mutations of genes *BRCA 1, 2*), oophorectomy is the only option for prevention. In general, clinical trials of cancer vaccines result in very modest efficacies in the range of 2.6% [12, 13].

Prophylactic vaccinations against many viruses and bacteria are very effective [14]. They are all organized by the Center for Disease Control (CDC) into the well-orchestrated schedule, which covers individuals of all ages from neonates through adulthood to elderly in the entire USA. The ability of their immune systems to protect against microbial insults are measured by the titers of antibodies and if necessary, when administered in denatured or synthetic form, can be easily reinforced by booster shots. Therefore, the immune systems of all persons in the USA, who are in compliance with the CDC scheduled vaccinations, are on the constant alert and capable for the instant response, which is promptly reinforced and amplified by their immune systems’ memory cells.

In particular, vaccines against Hepatitis B Virus (HBV) are approved by the FDA and recommended by the CDC to be administered as the first vaccine after birth. The currently approved vaccines are: Engerix B and Recombivax [15, 16]. Both are virus like particles of the human hepatitis B virus surface antigen (HBsAg). Mechanism of action of this vaccine relies upon activation of both arms of the immune system: innate and acquired. Therefore, exposure to the hot virus triggers complete, immediate and effective, humoral and cellular response annihilating the infecting HBV. Measure of the immune system readiness is production of antibodies by immune cells at the titers above 10.0 mIU/ml. If the antibody titer falls below that aforementioned value, the booster dose quickly reinvigorates the effective immunity. Thanks to this program in the USA, incidence of Hepatitis B declined 82% over 17 years, i.e., from 8.5 cases per 100,000 population in 1990 to 1.5 cases per 100,000 population in 2007.

The ultimate goal of our work is to engage the entire immune system (conditioned by the scheduled microbial vaccinations or acquired by natural infections to their full readiness) as the natural therapeutics of cancer. In this realm, we pursue designing, synthesis, and streamlining to clinical practice of antibody × vaccine engineered constructs (AVEC), which could redirect and engage prophylactic immunity from the original aim—protecting from viruses, towards the new one-curing cancers.

### Specific aim

The specific aim of this project was engineering of molecules capable of redirecting, accelerating, and amplifying immunity from the preventive immunity, attained due to HBsAg vaccination against HBVs, towards the therapeutic immunity against HER-2+ ovarian cancers.

## Patients’ biopsies

### Declaration of Helsinki compliance. Institutional Review Board approval. Patient informed consent

Tissue biopsies, ascites, and blood were acquired from the twelve patients suffering from the advanced ovarian cancers, from the patients suffering from acute and chronic infection with HBV, and from the healthy volunteers having high titers of antibodies induced by standard HBsAg vaccination. All the samples were acquired and all the data presented with the patients’ identity entirely concealed in accordance with the Declaration of Helsinki, pursuant to the Institutional Review Board approval, and with the Patients’ informed consent.

## Experimental design

**Fig. 1:**
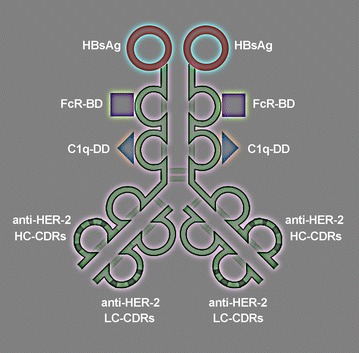
Functional, molecular architecture of AVEC: anti-HER-2 × HBsAg. The architecture of antibody vaccine engineered constructs (AVEC: anti-HER-2 × HBsAg) is shown. AVEC consist of the effector domains: HBsAg, FcR-BD, C1q-BD and the targeting domains: anti-HER-2 CDRs. HBsAg: human hepatitis B virus surface antigen vaccine; it is the hepatitis B virus like particle (VLP) vaccine, which engages native and stimulates development of adaptive arms of immunity, upon vaccination both arms are engaged as prophylactic immunity against the hot hepatitis B virus. FcR- BD: a crystallizable fragment of the antibody (Fc) is its binding domain for FcR receptors (FcR), i.e., a ligand for FcR; as it is thus anchoring all FcR displaying cells: natural killer cells, dendritic cells and after presenting antigens by APCs by cytotoxic lymphocytes (CTL), B lymphocytes, macrophages, neutrophils, basophils, eosinophils, mast cells, and others. C1q-BD: it is a docking site for C1q element of the complement system cascade (C1q) leading to its main effector C3 leading to assembly of the complex and perforation of the cell membranes. Anti-HER-2 CDR: human epithelial cell receptor 2 complementarity determining regions of light (anti-HER-2–LC) and heavy (anti-HER 2–HC CDR) chains of the antibody guide the AVEC to the overexpressed HER-2+ displaying cancer cells

The novel, immunity redirecting biomolecule (AVEC: anti-HER-2 x HbsAg) contains main functional domains: anti-HER-2 antibody (which contains epithelial growth factor receptor 2 (HER-2) targeting, antibody constant fragment receptor (FcR) binding, and C1q component of the complement docking (CD) domains; HBsAg, which is the virus like particle (VLP) of human hepatitis B virus (HBV VLP) (Fig. 1).

## Methods

### Human ovarian epithelial cancer (HOEC), ovary surface epithelial (HOSE), human artery endothelial (HAE) cells

Human epithelial growth factor receptor positive (HER-2+) human ovarian epithelial cancer cell lines—OV-90 (CRL-11732) and TOV-112D (CRL-11731) were from the American Type Culture Collection (ATCC, Rockville, MD, USA) [17, 18]. They were derived from the ascites of the advanced, metastatic ovarian adenocarcinoma grade 3, stage IIIc. It is her2/neu+ and p53 mutated. It is cultured in the base medium: 1:1 mix of MCDB105 and 199 with the final concentration of sodium bicarbonate 2.2 g/l. It is supplemented with 15% of human serum. It contains 100 units/ml penicillin, 200 mg/ml streptomycin, in the cell culture 75 cm^2^ flask (Corning) (catalog #430641) in incubators set at 5% CO_2_ at 37 °C. The medium is replaced every 3 days. To split, the cultures are briefly rinsed with 0.25% (w/v) Trypsin, 0.53 mM EDTA solution to remove all traces of serum which contains trypsin inhibitor and thereafter treated with that solution. After dispensing into new flasks, they are grown in the same conditions.

Human epithelial growth factor receptor positive (HER-2+) human ovarian epithelial cancer cell lines—SK-OV-3 (HTB-77) and OVCAR-3 (HTB-161) were from ATCC [19, 20]. They were derived from the ascites of the advanced ovarian adenocarcinoma. They were grown in McCoy’s Modified Medium supplemented with 10% human serum. The cells were grown in the cell culture 75 cm^2^ flask (corning) (catalog #430641) in incubators with 5% CO_2_ at 37 °C. The cultures were split as described above.

Human ovary surface epithelial (HOSE) cells were derived from ovaries removed by prophylactic oophorectomies [21]. The cells were transfected with SV40 to extend their life span. They carried BRCA 1, 2 mutations. They were cultured in the base medium: 1:1 mix of MCDB105 and 199 supplemented 15% of human serum, 0.25 U/ml of insulin, 2 mM of l-glutamine. The media also contained 100 U/ml penicillin, 200 mg/ml streptomycin. The cells were grown in the cell culture 75 cm^2^ flask (Corning) (catalog #430641) in the CO_2_ incubators at 37 °C. The cultures were propagated as described above.

### Biotechnology of anti-HER-2 and anti-HBsAg antibodies and biosimilars

Biotechnology of anti-HER-2 synthetic antibodies was pursued by adaptation of that originally described, either as new antibodies or as biosimilars to the FDA approved: trastuzumab and pertuzumab as the positive controls [8, 21–23]. For verification, the DNA plasmid constructs for the anti-HER-2 antibodies variable fragments were imported from the International ImMunoGeneTics (IMGT, Paris, F, EU) antibody sequences’ bank [8, 21–23].

Briefly, in the first technology, the B cells were isolated from the blood of patients suffering from the cancers. White blood cells (WBC) were isolated using Ficoll-Hypaque technique. The total mRNA was isolated using Trizol reagent (Molecular Research Center, Inc. Cincinnati, OH). The cDNA was generated using random hexamers (Intergrated DNA Technologies, Coralville, IA) and reverse transcriptase (Promega, Madison, WI) in reactions involving denaturing RNA at 70 °C followed by reverse transcription carried at 42 °C for 15 min. The cDNA quality was tested by the polymerase chain reaction (PCR) of beta actin and GAPDH as reference genes with the commercially available primers (ABI, Foster City, CA). For amplification of coding sequences of the variable fragments, the primers' sets were designed using the Kabat database. They were synthesized on 380A DNA Synthesizer (ABI, Foster City, CA). The variable fragments were amplified with polymerase chain reaction using the mix of the generated cDNA, the synthesized primers, dNTPs, and Taq DNA polymerase (Hoffmann–La Roche, Basel, Switzerland) on the Robocycler (Stratagene, San Diego, CA) or Mastercycler (Eppendorf, New York, NY). The blunt ended amplicons were inserted into the plasmid coding for the constant regions of the human antibodies with sequences imported from the Gene Bank. The DNA plasmid constructs also contained metal binding domains capable of chelating superparamagnetic and fluorescent metals as detailed [8]. After electroporation of plasmids into fresh B cells or cultured human myelomas, they were propagated and expressed.

For selection and in vitro evolution, the HER-2 receptors were extracted from the human HER-2+ ovarian cancer cells by immunoprecipitation of rapidly frozen, crushed, thawed, and lyophilized. Alternatively, the mimotopes of HER-2 were manufactured. Both served as the baits and references for validation of antibodies.

Alternatively, biotechnology of trastuzumab and pertuzumab biosimilars was crafted on such a way that the coding sequences for the anti-HER-2 antibodies’ variable fragments were imported from the IMGT. These sequences were synthesized, cloned, expressed, and modified on the same way as the newly developed anti-HER-2 antibodies as described above.

For generating of anti-HBsAg antibodies the B cell were acquired from the patients suffering from the Acute hepatitis B. The protocol was identical to that published [27]. Dane particles, isolated from the patients’ blood by PEG gradients precipitation or from liver biopsies by CsCl gradient centrifugations, were rapidly frozen, lyophilized and stored. Alternatively, HBsAg were produced in transfected with the plasmid DNA human hepatoma cells. Prior to selection, during in vitro evolution, they were reconstituted with buffer and served as the baits. They also served as the negative controls for anti-HER-2 antibodies.

The metal binding domains of the antibodies were saturated with Gd, Tb, Ru, Ni, Co, or Eu or linked with Au coated Fe_3_O_4_ (Au(Fe_3_O_4_)) nanoparticles. The specificity and sensitivity were determined based upon elemental spectra acquired with EDXS (Noran, Middleton, WI, USA), EELS (Zeiss, Oberkochen, D, EU), or TRXFS (Bruker AXS, Fitchburg, WI, USA). The fluorescent properties were measured with the RF-5301PC spectrofluorometer (Shimadzu, Tokyo, Japan). The magnetic relaxivities were measured on the DMX 400 WB or AVANCE II NMR spectrometers (Bruker Optics, Dallas, TX, USA).

### Biotechnology of HBsAg

HBsAg was isolated from the patients suffering from acute hepatitis B: either from the blood by PEG fractionations or from the liver biopsies by CsCl gradient centrifugation.

To assure exact immunogenic compatibility with the immunity induced by vaccinations with the FDA approved HBsAg, which were produced in yeast, the HBsAg in this project were also generated in yeast as originally described [8, 21–23]. Biotechnology of the recombinant HBsAg was pursued based upon the published DNA coding sequence [24, 25]. Hepatitis B virus like particles (VLP) were initially synthesized in yeast—*Saccharomyces cerevisiae* as originally described. In particular, the expression plasmid pHBS-16 included the HBsAg surface antigen (HBsAg) controlled by the yeast alcohol dehydrogenase (*ADHI*) promoter through introduced by EcoRI restriction sites into the DNA construct of the pBR322 plasmid. That followed by yeast replication origin, yeast trp1 gene. This biotechnology was later modified to be pursued in Pichia pastoris [24]. Briefly, yeast cultures of Pichia pastoris were grown at 30 °C in rich medium (YPD; 1% yeast extract, 2% bactopeptone, 2% glucose) initially and shifted either to synthetic media (YNM, 0.67% yeast nitrogen base supplemented with 0.5% (v/v) methanol) for immunoprecipitation and immunofluorescence experiments, or to mineral media (MMOT, 0.2% (v/v) oleate and 0.02% (v/v) Tween-40) for fractionation studies.

All the protocols’ products—HBsAg VLPs were referenced and validated to the FDA approved and the CDC recommended Engerix B and Recombivax and the anti-HBV antibody titer assays [15, 16].

### Biotechnology of fluorescent and superparamagnetic mimotopes

Design of HER-2 cyclic mimotopes was initiated by importing the DNA from the GenBank and in vitro translation into amino acid sequences or direct amino acid sequences from SwissProt into the Peptide 3D or LaserGene software. That followed by determination of surface displayed domains. Further, molecular computer aided modeling led to selection of the most likely immunogenic domains. The 12–40 amino acids long sequences were selected. The amino acid sequences were exported. The selected sequences were altered by introducing glycine linkers with terminal cysteines at both amino and carboxyl terminus of the peptide designs. The designed peptides were synthesized as linear on the peptide synthesizer. After detachment from the cartridges, the peptides were converted into cyclics by means of the cysteines. The synthetic products—HER-2 mimotopes were selected and validated on the high pressure liquid chromatography columns. The specificity of the mimotopes was validated by binding to trastuzumab and ant-HER-2 biosimilars with the aid of MACS or FACS.

### Biotechnology of anti-HER-2 × HBsAg biomolecular clusters

The synthetic anti-HER-2 antibodies and synthetic HBsAg VLPs were linked with heterospecific, bifunctional linker–sulfo-m-maleimidobenzoyl-N-hydroxysuccinimide ester (SMBS) after adapting the protocol [27]. Briefly, the anti-HER-2 antibody was dialyzed against 0.15 M sodium chloride, 0.1 M sodium phosphate, at pH 7.2. Sulfo-MBS stock in DMSO was added to this solution up to the final 2% w/v concentration to assure at least 80× molar excess. After 1 h at room temperature, the reaction solution was rapidly applied to desalting columns. Performing chromatography with the 0.15 M sodium chloride, 0.1 M sodium phosphate, at pH 7.2 carrier solution was followed by pooling the activated anti-HER-2 antibodies’ 1 ml fractions. To this solution, the synthetic HBsAg diluted in the same carrier solution was promptly added to assure 1:1 ratio. The reaction continued for 1 h at room temperature. The effective anti-HER-2 × HBsAg clusters were isolated by chromatography.

The specificity of the anti-HER-2 × HBsAg to label HER-2 receptors was validated by FCM, NMR and XRFS on cells and mimotopes. The specificity of the anti-HER-2 × HBsAg to attract immune response was validated by labeling with anti-HBsAg antibodies rendered fluorescent for FCM or superparamagnetic for NMR or element specific by XRFS [8, 21].

### Immunoblotting and immunoprecipitation

The cells and tissues were either frozen crushed in the rapid controlled rate freezer (the NSF grant support to MM).or native disintegrated with ultrasonicator (Branson Ultrasonic, Danbury, CT, USA). After being homogenized within the sample buffer they were either stored in liquid nitrogen or lyophilized. They were electrophoresed in the native buffer (Invitrogen, Carlsbad, CA, USA). They were vacuum- or electro-transferred onto the PVDF membranes (Amersham, Buckinghamshire, UK, EU). The membranes carrying the transferred proteins were first soaked within human serum and thereafter labeled with the bioengineered, biosimilar, and referenced anti-HER-2 antibodies. The anti-HBsAg isotype antibodies served as the controls. The images of the blots were acquired and quantified with Fluoroimager (Molecular Dynamics, Sunnyvale, CA, USA) or Storm 840 (Amersham, Buckinghamshire, UK, EU).

The anti-HER-2 and anti-HBsAg antibodies were rendered magnetic or fluorescent by conjugating Au coated Fe_3_O_4_ nanoparticles. The sera and liver biopsies’ homogenates were mixed with these superparamagnetic antibodies. The targeted molecules rendered superparamagnetic were pulled out by the means of 1.5T magnet. The intensity of fluorescence was measured on the spectrofluorometer to determine the concentration of HER-2 mimotopes of HBsAg VLPs.

### Fluorescent antibodies

#### Fluorescent, activated cell sorting

##### Flow cytometry

###### Multiphoton fluorescence spectroscopy

Ovarian cancer cells were labeled with the fluorescent antibodies. They were sorted on the Calibur, Vantage SE, or Aria (Becton–Dickinson, Franklin Lakes, NJ, USA). The antibodies were dissolved and all washing steps carried in phenol-free, Ca+/Mg+—free, PIPES buffered saline solution, supplemented with 20 mM glucose, 10% human serum. Sorting was performed on Aria, Calibur, Vantage SE (Becton–Dickinson, Franklin Lakes, NJ, USA) with the sheath pressure set at 20 lb per square inch and low count rate. The sorted batches were analyzed on Calibur or Aria using FACS Diva software or on the FC500 (Beckman-Coulter, Brea, CA, USA). For the measurement of the fluorescently labeled cells, these settings were tuned at the maximum emission for the Eu chelated antibody at 500 V with references to isotype antibodies and non-labeled cells. This assured the comparisons between populations of cells labeled with multiple antibodies without changing the settings on PMTs.

The fluorescently labeled cells or tissues were imaged with the Axiovert (Zeiss, Oberkochen, D, EU) equipped with the Enterprise argon ion (457, 488, 529 nm lines) and ultraviolet (UV) (364 nm line) lasers; Odyssey XL digital high-sensitivity with instant deconvolution confocal laser scanning imaging system operated up to 240 frames/s (Noran, Madison, WI, USA), and the Diaphot (Nikon, Tokyo, Japan) equipped with the Microlase diode-pumped Nd:YLF solid state laser (1048 nm line).

### Superparamagnetic antibodies

#### Nuclear magnetic resonance spectroscopy

##### Magnetic activated cell sorting

Ovarian cancer cells were labeled with the superparamagnetic anti-HER-2 and anti-phosphatidylserine (anti-PS) antibodies [8]. The antibodies were dissolved and all washing steps carried in phenol-free, Ca+/Mg+—free, PIPES buffered saline solution, supplemented with 20 mM glucose, 10% human serum. The aliquots were dispensed into the magnetism-free NMR tubes (Shigemi, Tokyo, Japan). The relaxation times T1 were measured in resonance to the applied pulse sequences on the NMR spectrometers: DMX 400 WB or AVANCE II NMR (Bruker, Billerica, MA*)* or the Signa clinical scanners (GE, Milwaukee, WI, USA). The superparamagnetic antibodies were also used to isolate the labeled cells from the solution. The cells labeled with the superparamagnetic antibodies were isolated on the magnetic, activated cell sorter operated at 1.5 T (the NSF grant support to MM).

### Elemental-tags modified antibodies

#### Energy dispersive x-ray spectroscopy

##### x-ray reflection fluorescence spectroscopy

The samples, which were cryo-immobilized, presented the life-like antigenicity and supramolecular organization. Elemental analyses were pursued by EDXS and XRFS as described (40). The field emission, scanning transmission, electron microscope FESTEM HB501 (Vacuum Generators, Kirkland, WA, USA) was equipped with the energy dispersive x-ray spectrometer (EDXS) (Noran, Middleton, WI, USA) and post-column electron energy loss spectrometer (EELS) (Gatan, Pleasanton, CA). The cryo-energy filtering transmission electron microscope 912 Omega was equipped with the in-column, electron energy loss spectrometer (EELS) and the energy dispersive x-ray spectrometer (EDXS) (Zeiss, Oberkochen, D, EU). The cryo-energy filtering transmission electron microscopes 410 and 430 Phillips were equipped with the post-column, electron energy loss spectrometers (EELS) and the energy dispersive x-ray spectrometer (EDXS) (Noran, Middleton, WI, USA). The field emission, scanning electron microscope SEM1530 (Zeiss, Oberkochen, D, EU) was equipped with the energy dispersive x-ray spectrometer (EDXS) (Noran, Middleton, WI, USA). The field emission, scanning electron microscope 3400 was equipped with the energy dispersive x-ray spectrometer (EDXS) (Hitachi, Tokyo, Japan). The S2 Picofox XRFS spectrometer was equipped with a molybdenum (Mo) x-ray target and the Peltier cooled Xflash Silicon Drift Detector (Bruker AXS, Fitchburg, WI, USA). Scan times ranged upto 1000 s. The ICP standard of 1000 mg/l of mono-element Gallium or Gadolinium (CPI International, Denver, CO, USA) was added to 500 microL of each sample to the final concentration of 10 mg/l. Instrument control, data collection, and analysis were under the SPECTRA 7 software (Bruker AXS, Fitchburg, WI, USA).

### Antibody-vaccine engineered construct-induced toxicity (AVECIT)

To study collective killing effects of the anti-HER-2 and anti-HER-2 × HBsAg upon the ovarian cancer cells, the patients’ cellular and serum fractions described below were pooled making erythrocytes-free blood (EFB). Anti-HER-2 and anti-HER-2 × HBsAg were added to the EFB. Similarly, anti-HBsAg, anti-HPV, and anti-EGFR1 antibodies were added as the controls. The incubation with the antibodies continued at the 37 °C incubators. The labeling continued for 1–24 h. It was terminated by washing with the cold buffer.

To quantify by flow cytometry (FCM) and fluorescent activated cell sorting (FACS) the numbers of killed cells, the samples were stained with propidium iodide (PI) (Sigma-Aldrich, Milwaukee, WI, USA) at 50 µg/ml. To determine the numbers of apoptotic cells, they were labeled with anti-phosphatidylserine antibodies or annexin.

### Antibody dependent cell cytotoxicity (ADCC)

To study toxicity to the cancer cells caused by the patients’ cytotoxic cells—the effectors triggered by the anti-HER-2 antibodies and AVEC, the peripheral blood mononuclear cells were separated from the blood on Ficoll-Hypaque density gradients. The cells were washed by three cycles of spinning down and suspending in the PBS at pH 7.3. They were rendered fluorescent by adding the stock solution of the DiI membrane dye (Molecular Probes, Inc., Eugene, OR, USA) in DMSO for 10 min at 26 °C. Small aliquots were washed with the buffer and the cells quantified on FCM as the way to determine the effector to target cells’ ratios (ETR). These ratios varied: 10:1, 50:1, and 100:1. Incubations lasted 1–24 h in a 37 °C, 5% CO_2_ incubator.

The numbers of killed cells were determined due to staining with the PI at 50 µg/ml or anti-dsDNA, apoptotic with anti-PS or annexin, and of surviving cells from the DiO staining cell counts.

### Complement dependent cytotoxicity (CDC)

To study toxicity to the cancer cells caused by the patients’ complement system—the effector triggered by the anti-HER-2 antibodies and AVEC, the serum was separated by gentle centrifugation from the freshly drawn blood. It was supplemented with the anti-HER-2 and anti-HER-2 × HBsAg. Incubations lasted 1–24 h in a 37 °C, 5% CO_2_ incubator. The numbers of killed cells were determined due to staining with the PI at 50 µg/ml apoptotic with anti-PS or annexin, and of surviving cells from the DiO staining counts.

### Statistical analysis

All the measurements were run in triplicates for each sample from six patients. The numbers were analyzed and displayed using GraphPad software (GraphPad Software, Inc, La Jolla, CA). Data were presented as mean of standard error of the mean (SEM). Statistical significance was calculated by *t* test for two groups (trial vs control).

## Results

### Sensitivity and specificity of anti-HER-2 × HBsAg in targeting HER-2^+^ ovarian cancer cells

**Fig. 2:**
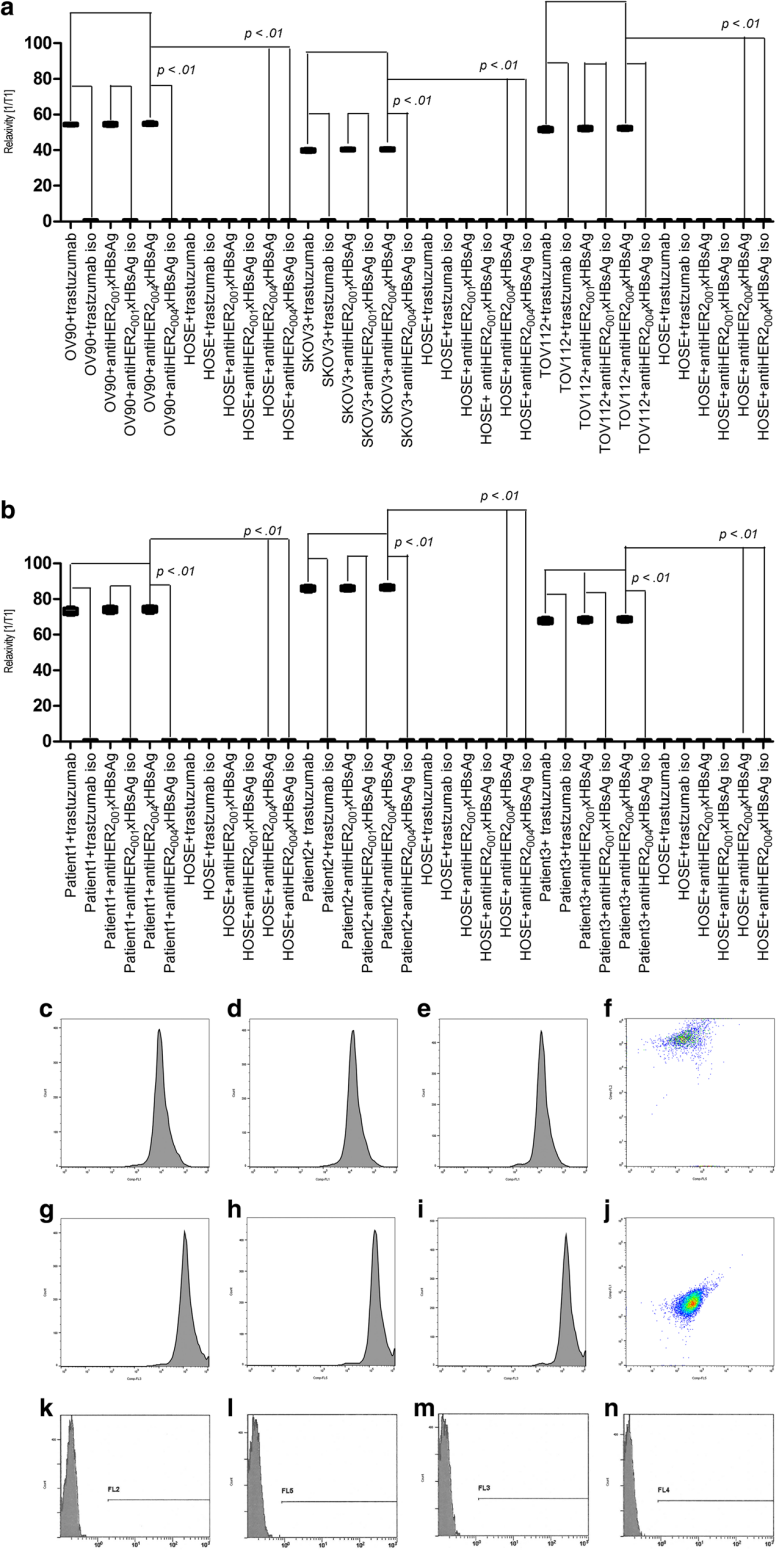
Sensitivity and specificity of AVEC: anti-HER-2 × HBsAg in targeting ovarian cancer cells. (**a**) The ovarian cancer cells from cultures (OV90, TOV112, SK-OV-30), from the ovarian epithelial cancers of the patients (001-0012), human ovary surface (HOSE), human artery endothelial (HAE) cells were labeled with trastuzumab, anti-HER 2001 × anti-HBsAg, anti-HER-2004 × anti-HBsAg, and relevant isotype antibodies rendered superparamagnetic. Antibodies labeling the cells were changing the cells’ magnetic properties, which were measured with nuclear magnetic resonance (NMR). The assays were repeated four times. The data presented are representative for all acquired. The changes in the length of T1 resulted from the changes in magnetic resonance, which were altered by presence of superparamagnetic antibodies and were proportional to the numbers of antibodies attached to the labeled cells. With the same number of cells in each batch, the relaxivity changes were directly proportional to the numbers of cell receptors displayed by the cells. Therefore, they facilitated comparisons of sensitivity of labeling between the cells, while being pursued with different antibodies. The OV90, TOV112, and SKOV3 cells were labeled with trastuzumab, anti-HER-2 biosimilars, and AVEC: anti-HER-2 × HBsAg at the statistically significant superiority over those labeled with the isotype antibodies and isotype-based AVEC. The control HOSE and HAE cells were labeled at the same levels as the isotype antibodies. (**b**) The ovarian cancer ascites cells from the patients vHBV001-012 were labeled with the same superparamagnetic antibodies, followed by the measurement of relaxivities, as outlined for cultured cells in **a**. The ovarian epithelial cancer ascites cells were all showing high numbers of the HER-2 receptors specifically labeled by the tested antibodies. Measurements of the relaxivities demonstrated statistically significant difference in the numbers of receptors on the ovarian epithelial cancer ascites cells over HOSE and HAE. Measurements of the relaxivities demonstrated statistically significant difference attained by labeling with the anti-HER-2 antibodies and AVEC over the relevant isotypes. The assays were repeated four times. The data presented are representative for all acquired (*i* isotype antibody). (**c**–**f**) The ovarian epithelial cancer cells from cultures (OV90, TOV112, SK-OV-30) and (**g**–**j**) the ovarian cancer cells from the patients 001-0012, (**k**–**n**) human ovary surface epithelial (HOSE), human artery endothelial (HAE) cells were labeled with trastuzumab (**c**,** g**), anti-HER-2001 × HBsAg, anti-HER-2004 × HBsAg (**h**,** k**), and relevant isotype antibodies (**k**–**n**) rendered fluorescent to acquire fluorescent properties. They were studied with flow cytometry (FCM) and fluorescent activated cell sorting (FACS). The fluorescently labeled ovarian cancer cultured cells facilitated their high counts and efficient sorting. The counts from the cells labeled with therapeutic antibodies and AVEC were statistically significantly higher than the cells labeled with the relevant isotype antibodies. These counts were statistically significantly higher than of HOSE and HAE cells. The assays were repeated four times. The data presented are representative for all acquired (*i* isotype antibody)

The most essential factors for attaining high efficacy of targeted immunotherapy of cancers is its specificity and sensitivity in targeting the receptors on cancer cells, while eliminating, or at least reducing, labeling of healthy cells (Fig. 2).

We quantified sensitivity in detection of HER-2 on cancer cells by labeling cells with superparamagnetic antibodies and measuring relaxivities by nuclear magnetic resonance (NMR). For this purpose, we labeled the OV-90, TOV-112D, SK-OV-3, which highly overexpress HER-2 (Fig. 2a). Moreover, we labeled the cells of the patients diagnosed with HER-2+ epithelial ovarian cancers (Fig. 2b). All these cells were labeled with anti-HER-2_001_, anti-HER-2_004_, AVEC: anti-HER-2_001_ × HBsAg, and anti-HER-2_004_ × HBsAg. As the control, the HOSE cells, which express HER-2 at the basal level, were labeled with the same antibodies. As the control, the human artery endothelial (HAE) cells were labeled with these antibodies. As the control, all these cells were also labeled with the isotype antibodies. As the reference controls, all these cells were labelled with trastuzumab and pertuzumab. HER-2^+^ OV-90, TOV-112D, SKOV-3 were heavily labeled with: trastuzumab, anti-HER-2_001_, anti-HER-2_004_, anti-HER-2_001_ × HBsAg, and anti-HER-2_004_ × HBsAg, as compared to HOSE and HAE cells. The differences were statistically significant. These measurements revealed also statistical differences between these novel therapeutics and the isotype antibodies. Therefore, we validated targeting of the HER-2^+^ ovarian cancer cells by the anti-HER-2 × HBsAg AVEC as highly specific and sensitive.

We determined specificity and sensitivity of AVEC in targeting HER-2 domains on the cultured cell lines by flow cytometry (FCM) and fluorescently activated cell sorting (FACS) (Fig. 2c–e). Moreover, we determined sensitivity and specificity by FCM and FACS after labeling patients’ cells (Fig. 2f–j). The FCM data corroborated with the NMR data. The HER-2+ overexpressing ovarian cells were effectively distinguished and sorted based upon fluorescently modified antibodies from the HOSE and HAE cells serving as the controls. These measurements revealed statistically significant difference between the ovarian cancer and healthy cells. As the reference controls, we labeled all these cells with trastuzumab and pertuzumab. As the controls, we also labeled all these cells with isotype antibodies. These measurements revealed statistically significant difference between the ovarian cancer cells labeled with anti-HER-2 antibodies and AVEC over isotype antibodies.

We performed tests of cross-blocking (Table 1). While labeling with anti-HER-2_001_ × HBsAg interfered on the statistically significant level with trastuzumab. However, labeling with anti-HER-2_004_ × HBsAg did not interfere with trastuzumab. Therefore, we concluded that trastuzumab and anti-HER-2_001_ × HBsAg target the same, but trastuzumab and anti-HER-2_004_ different domains on the HER-2 receptors. Moreover, neither trastuzumab, nor anti-HER-2 had any impact on binding of anti-HBsAg. However, AVEC: anti-HER-2 × HBsAg was effectively competing with HBsAg for binding of anti-HBV/anti-HBsAg.

**Table 1:** Cross blocking of antibodies

	Trastuzumab	Anti-HER-2_001_	Anti-HER-2_004_	Anti-HER-2_001_ **×** HBsAg	Anti-HER-2_004_ **×** HBsAg	Anti-HBsAg
Trastuzumab	+	+	–	+	–	–
Trastuzumab i	–	–	–	–	–	–
Anti-HER-2_001_	+	+	–	+	–	–
Anti-HER-2_001_i	–	–	–	–	–	–
Anti-HER-2_004_	–	–	+	–	+	–
Anti-HER-2_004_ i	–	–	–	–	–	–
Anti-HER-2001 × HBsAg	+	+	+	+	+	+
Anti-HER-2_001_ × HBsAg i	–	–	–	–	–	–
Anti-HER-2_004_ × HBsAg	–	–	–	–	–	+
Anti-HER-2_004_ × HBsAg i	–	–	–	–	–	–
Anti-HBsAg	–	–	–	–	–	+
Anti-HBsAgi	–	–	–	+	+	–

### Sensitivity and specificity of anti-HER-2 × HBsAg to attract anti-HBV antibodies and cells

**Fig. 3:**
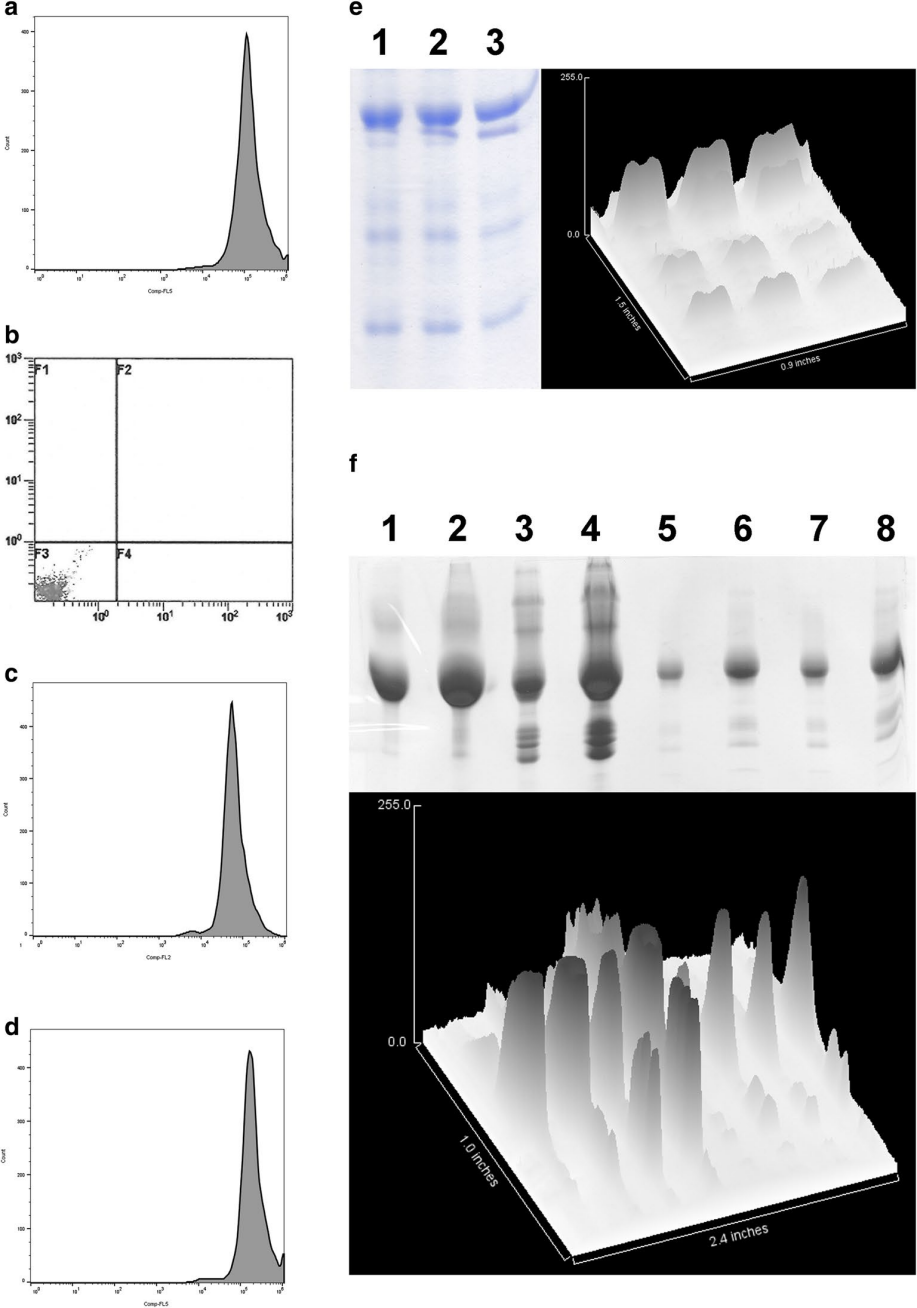
Sensitivity and specificity of AVEC: anti-HER-2 × HBsAg to attract anti-HBV antibodies. The ovarian cancer cells from cultures (OV90, TOV112, SK-OV-3) and from the patients (001-0012), human ovary surface (HOSE), human artery endothelial (HAE) cells were labeled initially with: (**a**) trastuzumab followed by the anti-FcR-BD fluorescent antibody, (**b**) trastuzumab followed by anti-HBsAg fluorescent antibody, (**c**) AVEC: anti-HER-2 × HBsAg followed by the anti-FcR-BD fluorescent antibody, (**d**) AVEC: anti-HER-2 × HBsAg followed by anti-HBsAg fluorescent antibody. The assays were repeated four times. The data presented are representative for all acquired. These cells revealed having high number of HER-2 receptors (>2 × 10^6^/cell) were effectively labeled with both trastuzumab and AVEC (**a**,** c**). However, only the cells labeled with AVEC attracted the anti-HBsAg antibody onto the ovarian cancer cells (**d**), but the cells labeled with trastuzumab did not (**b**). (**e**) Blood from the patients (V-HBV-001-003) vaccinated against HBV were depleted of erythrocytes, magnetic AVEC’s: anti-HER-2 × HBsAg were used to pull out anti-HBsAg, while the concentrations were adjusted to 10.0 mIU/ml or as in those administered in the forthcoming experiments, and electrophoresed. The gels were quantified. These data show very specific and sensitive affinity of the patients’ anti-HBsAg towards AVEC. Some of the antibodies were undergoing some degree of chain separation showing up as faster bands. (**f**) Blood from the patients vaccinated against HBV were depleted of erythrocytes, magnetic AVEC’s: anti-HER-2 × HBsAg were used to pull out anti-HBsAg and electrophoresed without adjusting the concentrations. The gels were quantified. The quantifications revealed different concentrations of the anti-HBsAg in different patients and different degree dissociation of the chains. The assays were repeated four times. The data presented are representative for all acquired

The most essential factor in engaging prophylactic immunity is specificity and sensitivity of the AVEC, anchored to the ovarian cancer cells, to bind the patients’ anti-HBV antibodies gained after vaccination with the VLP for HBV (Fig. 3).

We determined AVEC’s specificity and sensitivity towards the patients’ anti-HBsAg by flow cytometry (FCM) and fluorescently activated cell sorting (FACS) (Fig. 3a–d). The ovarian culture cells were labeled with antibodies against HER-2 highlighted with fluorescein or Eu or Tb. That followed by labeling with the anti-HBsAg highlighted with rhodamine or Tb. The anti-HBsAg from the vaccinated patients were only tagging the ovarian cancer cells labeled with AVEC: anti-HER-2 × HBsAg. However, they were not tagging the ovarian cancer cells labeled with trastuzumab or anti-HER-2 biosimilars.

We quantified AVEC’s specificity and sensitivity towards the patients’ anti-HBsAg by immunoprecipitation of the patients’ anti-HBsAg antibodies with superparamagnetic AVEC and HBsAg mimetics followed by electrophoresis (Fig. 3e, f). The concentration of the anti-HBsAg antibodies was adjusted to the concentrations indicated for studies of the factors affecting ADCC and CDC (Fig. 3e). Alternatively to the standard clinical assays, the anti-HBsAg quantities and profiles were determined through immunoprecipitation followed by unadjusted runs (Fig. 3f).

The most significant outcome of these tests was the demonstration that the AVEC, through CDRs—can at the same time very specifically bind to the ovarian cancer cells and through HBsAg—very specifically attract to these ovarian cancer cells the antibodies of the vaccination induced adaptive immunity. By extrapolation, this would also indicate attracting the elements of the innate immunity, as the HBV would do.

### Mechanism of action of anti-HER-2 × HBsAg in ovarian cancer cells’ killing

**Fig. 4:**
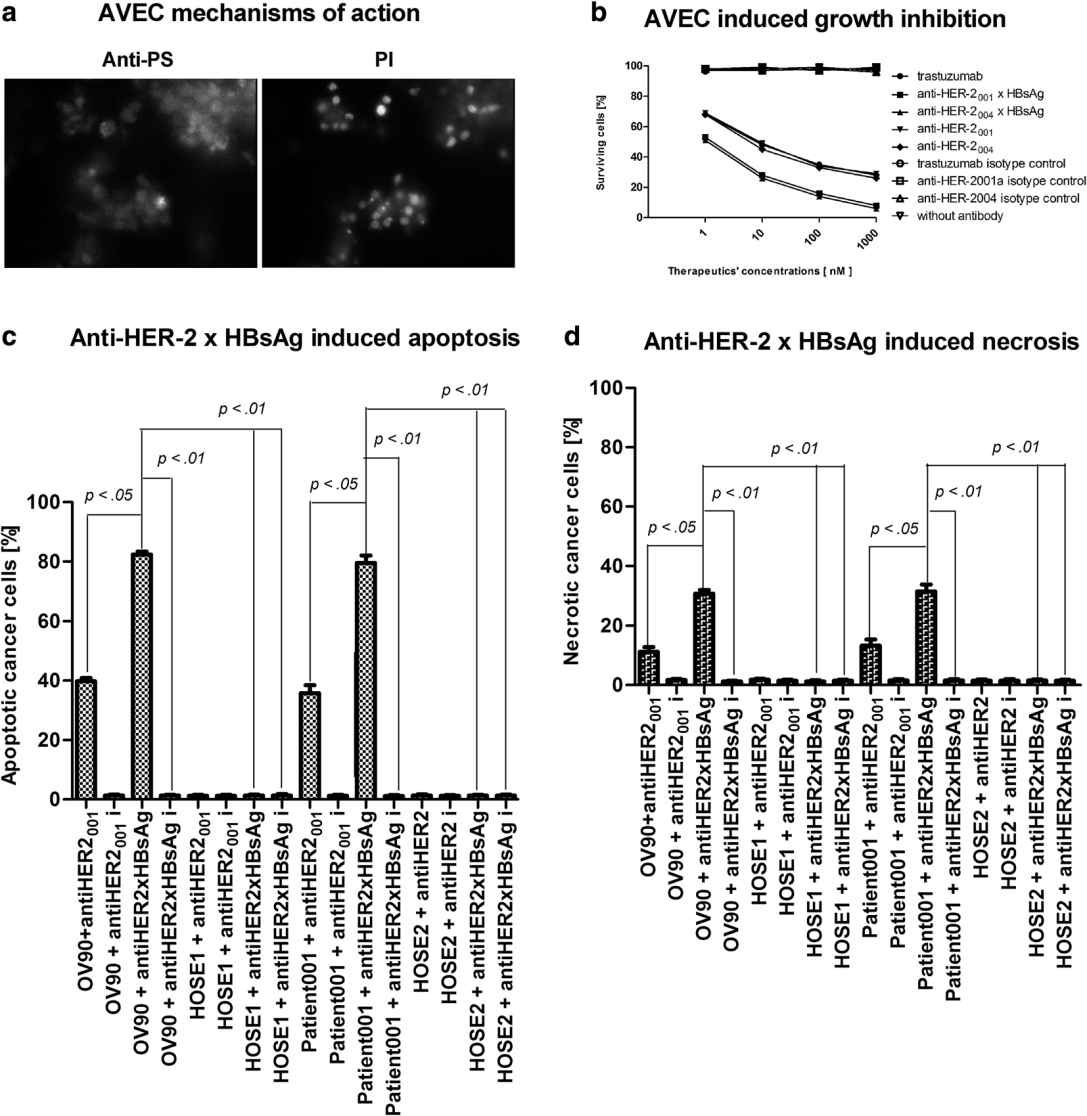
Mechanism of action of AVEC: anti-HER-2 × HBsAg in ovarian cancer cells’ killing. (**a**) The patients’ ovarian cancer cells were labeled for 6 h at 37 °C with 0.3 mg/ml trastuzumab or anti-HER-2 × HBsAg (B) in full erythrocytes’ free blood from the HBV-vaccinated patients with the anti-HBV adjusted to 10.0 IU/ml. That was followed by labeling with anti-phosphatidylserine (anti-PS) or propidium iodide (PI) or anti-genomic DNA (antigDNA). While most of the cells demonstrate a flip of phosphatidylserine, only some of them have compromised membranes’ permeability for makers of intranuclear, genomic DNA. The assays were repeated four times. The data presented are representative for all acquired. (**b**) Effects of AVEC upon the ovarian cancer cells’ growth was studied by incorporation of tritium tagged thymidine. AVEC inflicted statistically significant higher impact upon the cells’ growth, when compared to isotype antibodies. AVEC had negligible effects upon HAE and HOSE cells. (**c**) Treatment induced apoptosis was evaluated by labeling with anti-phosphatidylserine (anti-PS) superparamagnetic antibodies and measuring relaxivity in NMR. Anti-HER-2 naked antibodies resulted in approximately 40% of cells being apoptotic. Treatment with the AVEC resulted in the number of apoptotic cells more than doubled. (**d**) Treatment induced necrosis was evaluated by labeling with anti-genomic DNA (anti-gDNA) superparamagnetic antibodies and measuring relaxivities in NMR. Anti-HER-2 naked antibodies resulted in approximately 10% of cells being necrotic. Treatment with the AVEC resulted in the number of necrotic cells nearly tripled.* I* isotype antibody. These assays were repeated four times. The data presented are representative for all performed

The three main mechanisms of action (MOA) attributed to immunotherapy are: inhibition of cell proliferation, apoptosis, and necrosis. Each of these MOAs was tested by fluorescence microscopy (Fig. 4a), NMR, and FCM by using the markers specific propidium iodide (PI) or anti-dsDNA and anti-phosphatidylserince or annexin. We performed the tests, each in triplicates, validating each of these mechanisms of action with regard to AVEC (Fig. 4).

The Impact on ovarian cancer cells proliferation was tested by pulsing with tritium marked thymidine (3H-T) following treatment with increasing concentrations of trastuzumab, anti-HER-2 biosimilars, and AVEC. The inhibition was calculated as percentage of cells incorporating thymidine as the sign of proliferation in the cells treated with AVEC or antibodies as compared to non-treated cells or cells treated with isotype antibodies as the controls. Growth inhibition was attained at much lower molar concentrations of AVEC: anti-HER-2_001_ × HBsAg and anti-HER-2_004_ × HBsAg, than those attained with naked antibodies: trastuzumab, anti-HER-2_001_, anti-HER-2_004_ (Fig. 4b).

Triggering apoptosis of ovarian cancer cells by trastuzumab, anti-HER-2 biosimilars, and AVEC was tested by quantifying phosphatidylserine externalization detected in flow cytometry and fluorescence microscopy. Anti-HER-2_001_ × HBsAg and anti-HER-2_004_ × HBsAg induced apoptosis in much greater percentage than trastuzumab (~40%) and or its biosimilars. AVEC and naked antibodies triggered apoptosis in statistically much higher rate in cancer cells than in HOSE and HAE cells treated on the identical way. AVEC triggered apoptosis at the statistically significant rate over the isotype antibodies (Fig. 4c).

Inducing necrosis in ovarian cancer cells by trastuzumab, anti-HER-2 biosimilars, and AVEC was tested by quantifying accessing the intranuclear DNA with flow cytometry and fluorescence microscopy. Anti-HER-2 antibodies and AVEC caused necrosis. At the initial stages of apoptosis, many cells were only showing outer-membrane display of phosphatidylserine, but were not permeable for propidium iodide (PI) or anti-dsDNA. However, the ovarian cancer cells quickly progressing through apoptosis were becoming leaky; thus adding to necrotic counts. AVEC caused massive necrosis of all HER-2+ ovarian cancer cells in the percentages, which were statistically significantly much higher than those inflicted by naked trastuzumab and biosimilars (Fig. 4d).

### Factors affecting immunotherapeutic efficacy of anti-HER-2 × HBsAg

**Fig. 5:**
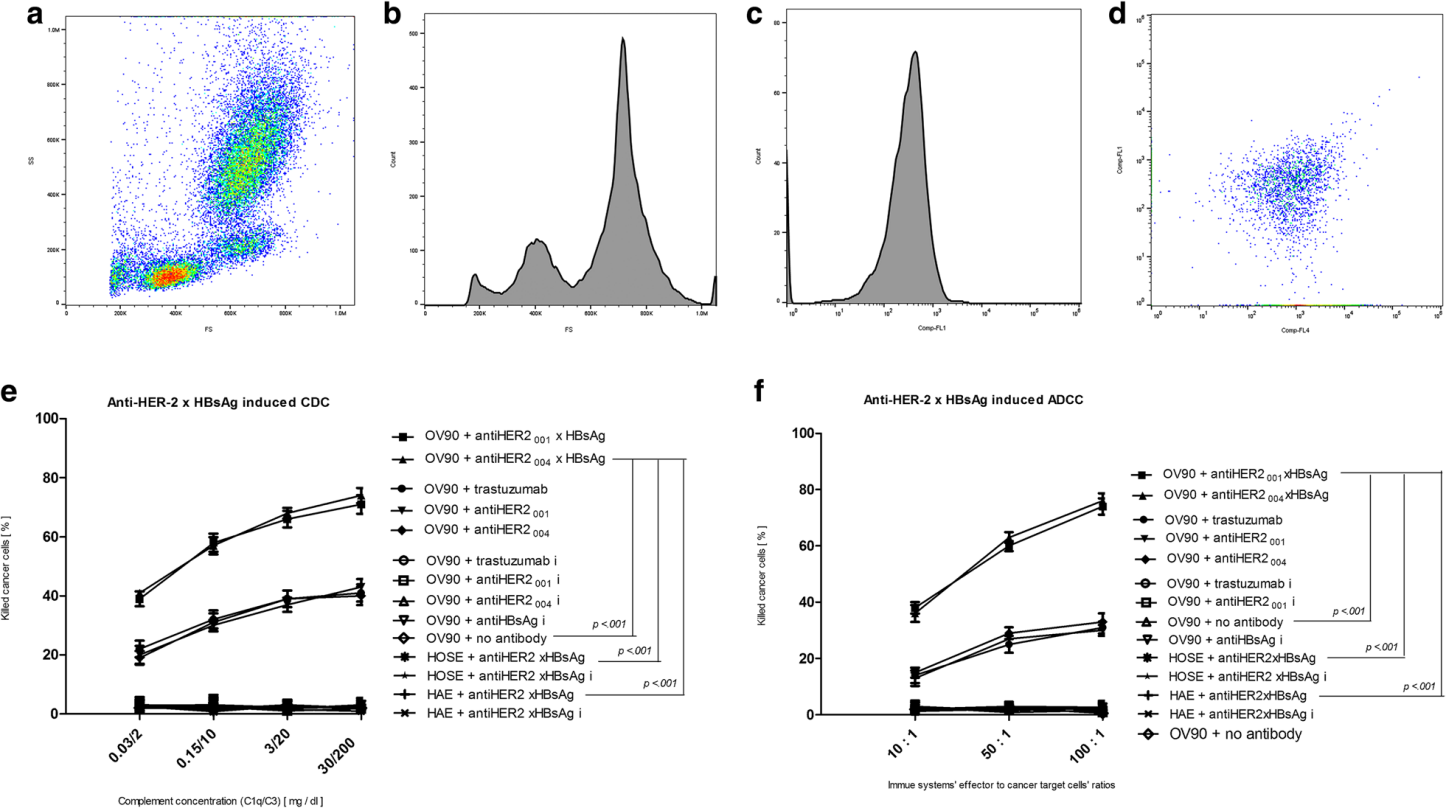
Factors affecting immunotherapeutic efficacy of AVEC: anti-HER-2 × HBsAg. (**a**–**d**) Blood of the HBV vaccinated patients was depleted of erythrocytes and analyzed. Three main populations of cells were revealed by forward and side scattering (**a**). Fractions of cells were determined through cell counts (**b**). Various fractions of white blood cells (WBC) were sorted based upon clusters of differentiations’ display, so that the number of cells and inter-fractions’ ratios could be adjusted in the forthcoming experiments. The OV90 cells were labeled with anti-HER-2 × HBsAg followed by anti-Fc-R-BD and anti-HBsAg (**d**). These assays were repeated four times. The data presented are representative for all performed. (**e**) OV90 cells were treated at 37 °C with trastuzumab, biosimilar anti-HER-2, anti-HBV, and anti-HER-2 × HBsAg in erythrocyte-free blood from the HBV vaccinated patients, while concentrations of the complement system were adjusted for C1q and C3 as indicated on the diagram. Increasing concentrations of complement system components resulted in increased efficacy of the ovarian cancer cells killing as complement dependent cytotoxicity (CDC). Importantly, treatment with AVEC at nearly three times lower concentrations of C1q and C3 resulted in nearly the same therapeutic efficacy as naked anti-HER-2 antibodies at three times higher concentrations. This efficacy was at significantly higher statistical rate than with the relevant isotype antibodies. This impact onto cancer cells was also of statistical significance difference over that onto HOSE and HAE cells. These assays were repeated four times. The data presented are representative for all performed.* I* isotype antibodies. (**f**) OV90 cells were treated at 37 °C with trastuzumab, biosimilar anti-HER-2, anti-HBV, and anti-HER-2 × HBsAg in erythrocyte-free blood from the HBV vaccinated patients, while the ratios between cytotoxic effector cells and the ovarian cancer cells were adjusted as indicated on the diagram. Increasing the ratio of the effector cytotoxic cells to ovarian cancer cells clearly increased efficacy of killing cancer cells by antibody dependent cytotoxic cells (ADCC). Importantly, ratios of effector to cancer cells, when AVEC were administered, resulted in the same immunotherapeutic efficacy as compared to higher ratios when naked antibodies were administered. In other words less cytotoxic cells were needed for AVEC to deliver the same therapeutic effect as more cells when naked antibodies were administered. This feature is critically important, when the patients are immunocompromised after the rounds of systemic therapy and the patients’ ability grow the immune cells is annihilated by intended to suppress proliferation of cancer cells, but as side effects universally suppressing proliferation of all patients’ cells. The AVEC’s efficacy was at statistically significant advantage over the relevant isotype antibodies. The AVEC’s impact onto cancer cells was also of statistically significant difference over that onto HOSE and HAE cells. These assays were repeated four times. The data presented are representative for all performed. * I* isotype antibodies

The processes of ovarian cancer cells’ deaths are triggered by the specific elements of the patients’ immune system: humoral and cellular, which lead to antibody dependent cell cytotoxicity (ADCC) and complement dependent cytotoxicity (CDC). We aimed at defining the main factors triggering them. In particular, we were focused on the effects of the complement concentrations and the effector cells to target cells ratios (Fig. 5).

For the aforementioned purpose, blood profiles were quantified by flow cytometry after removal of the red blood cells (Fig. 5a, b, d). Various classes of the white blood cells were sorted and quantified by out by FACS (Fig. 5c). The concentrations of complement’s components were also determined by spectroscopy. These were the starting conditions to quantify ADCC and CDC.

Numbers of natural killer cells and cytotoxic lymphocytes determine this patients’ ability to execute antibody dependent cell cytotoxicity (ADCC). The numbers of the immune cells were adjusted to clinical lab values 10,000 cells/ml. Trastuzumab and our anti-HER-2 biosimilar antibodies caused the cancer cells’ deaths through ADCC at the ratio of 10:1, while with no statistical difference between them. Anti-HER-2_001_ × HBsAg, and anti-HER-2_004_ × HBsAg inflicted, through ADCC, massive deaths of ovarian cancer cells, at much lower ratios. Furthermore, the AVEC triggered ADCC resulting in the ovarian cancer cells’ deaths at statistically significantly much higher percentages, than those inflicted by trastuzumab and anti-HER-2 biosimilars. Treatment of the HOSE cells and human artery endothelia (HAE) cells did not have any statistically significant impact on their death rates. Treatment with the isotype antibodies did not have any statistically significant impact on the HER-2+ epithelial ovarian cancer and healthy cells’ death rates.

These quantifications, demonstrated evidence of anti-HER-2_001_ × HBsAg, and anti-HER-2_004_ × HBsAg having statistically great statistical difference in inflicting cancer cells’ death over that on the HOSE and HAE cells. This characteristic is essential for reducing potential side effects of this immunotherapy on healthy ovarian cells and endothelial cells of the patients.

C1q is the first element of the patients’ complement system, which initiates the CDC cascade. The concentrations of the complement systems’ components, in particular C3, determine the patients’ ability to fight cancer by CDC. Concentrations of the complement systems’ components in our tests were adjusted within the ranges of healthy adults. Our measurements revealed that the increasing the concentrations of the C1q and C3 resulted in the statistically significant increase in the efficacy of the HER-2^+^ OV-90, TOV-112D, and OVCAR-3, and the patients’ epithelial ovarian cancer (EOC) cells killing by AVEC, trastuzumab, and anti-HER-2 biosimilar antibodies as compared to labeling with the isotype antibodies. The identical labeling of HOSE and HAE cells did not have any statistically significant impact upon them. This efficacy was statistically significantly much higher, when the HER-2^+^ EOC cells were treated with anti-HER-2_001_ × HBsAg, and anti-HER-2_004_ × HBsAg than with trastuzumab and anti-HER-2 antibodies.

## Discussion

The main result of this work is greatly improved efficacy and selectivity in cancer cell killing by antibody-vaccine engineered constructs (AVEC) over naked antibodies. AVEC create a novel paradigm for therapy of cancer as redirecting, accelerating, amplifying vaccine-induced immunity therapy (RAAVIIT).

Redirecting of the patients’ immunity is guided by HBsAg integrated into one molecule with anti-HER-2. Therefore, immune system response against HBV is redirected towards HER-2 and through that receptor against HER-2+ displaying cancer cells. This immune response consist of both branches of immunity: innate—as the innate response to the HBV and acquired—as the acquired immunity recognizing the HBV through its HBsAg used the vaccine. As the consequence, humoral and cellular effectors of the immune system become involved. This is a paramount advantage over using only a fraction of the immune system offered by a dose of passive immunity—naked monoclonal antibodies.

Amplifying of the patients’ immune response results from the presence of multiple domains of the HBV contained within HBsAg. Therefore, memory cells carrying the codes for polyclonal antibodies targeting multiple domains of the HBsAg produce multiple clones of antibodies, while all targeted towards the same cancer cells tagged by the variable fragments of AVEC. This is a significant amplification of the immunotherapeutic effect over monoclonal antibody targeting single one domain only. The cells activated by HBsAg produce cytokines responsible for amplification of the immune system and systemic response to therapy. Furthermore, antigen presenting cells and released cytokines induce production of more antibodies against HBsAg. Since, HBsAg is anchored on cancer cells, the newly produced antibodies keep coming to these cells, attract cytotoxic cells, and perpetrate cancer cell killing. Moreover, continuously amplified processes of production of the antibodies continue as long as there is HBsAg stimulation, while these newly produced antibodies are utilized by AVEC to trigger cancer cell killing. This is another very important advantage of the active immunity strategy presented here over passive immunity delivered by the single clone of the therapeutic monoclonal antibody.

Immunity developed in patients by vaccination relies upon generating polyclonal antibodies. Development of two immunotherapeutics: trastuzumab and pertuzumab, against different domains of the same receptor the same target—HER-2, is de facto an attempt of reconstructing the response of the natural immune system with polyclonal antibodies. Herein, we present a way to by-pass the need for developing multiple clones of antibodies against HER-2, but rather we use HBsAg, which serves as a lightning rod for attracting all the clones of antibodies generated by immunization. Therefore, it amplifies the therapeutic efficacy of the single clone of anti-HER-2 to the level equivalent to eliciting polyclonal antibodies. With this new strategy, we engage the multiple clones of the entire immune system against cancer targets; thus greatly amplify the therapeutic efficacy.

Acceleration of the response, when comparing timing of response yielded by AVEC versus developing immunity after eruption of cancer is significantly faster with AVEC. Thanks to the CDC scheduled and the FDA approved HBV vaccination, the entire immune system has already passed the learning process and has attained functional ability, when the patient was with good health. Sometimes, it may require the booster shots. Building that immunity, it all took time and energy. With the patients suffering from cancers, the time works against them. Moreover, they are often exhausted by the rounds of proliferation suppressing drugs, which as their side effects obviously suppress proliferation of immune cells. Therefore, the time is of essence and every minute counts. In this realm, having immune system, which is already conditioned, offers an important advantage over using the passive immunity or starting cancer vaccination for the cancer, which is already in progress and is hijacking all nutrients for its progression.

The primary condition for reaching this high therapeutic efficacy is the perfect match between two molecular interfaces: (I) complementarity determining region of the anti-HER-2 antibody and the antibody docking domain of the HER-2 receptor; (II) the complementarity determining region of anti-HBsAg antibody and the antibody hosting domain of the HBsAg.

The tests required by the FDA, which have to be performed prior to making the therapeutic qualification decision, are performed by immunocytochemistry on de-paraffinized sections. Denaturation of HER-2 during these harsh procedures may change the conformation of the epitope and result in false results. Moreover, the antibodies have different specificities and sensitivities for native versus denatured proteins. Furthermore, any variants in the amino acids sequence, result in mistargeting of the therapeutics. Therefore, the very thorough companion diagnostic evaluation of molecular arrangement of the HER-2 on heterogenic populations of cancer cells is essential.

The FDA approved vaccines and tests determining the concentrations of the antibodies in blood of the patients were against adw type of HBV. The interfacing molecules described herein are utilizing the patients’ immunity against those molecules. However, vaccinations against other types of the viruses or viruses’ mutations may result in deviations of reading of the adw oriented tests from the real levels of immunity [26, 27]. Identifying, the specific virus type used for vaccination of the particular patient, administering the specific tests, and applying the specific HBsAg, should be the essential part of designing therapeutic regimens.

Two strategies of therapy for ovarian cancer, which are currently promoted through in vitro experiments, are worth discussing in the context of this work: targeting cytolytic viruses by bispecific antibodies and recombinant oncolytic viruses [28, 29].

In experimental viral therapies, while most of the hot, e.g., Measles cytolytic viruses are anchored to the targeted cancer cells with specific antibodies and/or DARPINs, some of them may become unbound or mistargeted. These are fully pathogenic viruses, which are fully capable of infecting healthy cells, propagation, and causing the eruption of serious diseases. Alternatively, for persons being in compliance with the CDC guidelines and vaccinated with the FDA approved vaccines, the therapeutically administered viruses are going to be annihilated by the acquired immunity.

While recombinant, e.g., Herpes virus is primarily targeted to the cancer cells after adhesion to cell surface receptors, its surface molecules have vast number of surface domains facilitating adhesion and entry into the cells by alternative paths. Alternatively, with the wide spread of Herpes, its carriers have high titers of antibodies, what we detect diagnostically.

Therefore, the above strategies may not be considered for the practical clinical applications. The novel therapeutic paradigm described herein, does not carry the aforementioned risks of inflicting iatrogenic damages, as the currently explored strategies involving oncolytic viruses do.

Reducing iatrogenic injuries to the patients is essential. In this novel paradigm of cancer therapy, the statistically significant difference in killing ovarian cancer cells versus healthy human ovarian surface epithelial and artery endothelial cells is of paramount importance for pursuing clinical trials. It allows us to maximize the therapeutic efficacy attained with AVEC onto cancer cells, while minimizing iatrogenic effects upon healthy cells. This difference offers critical advantage over currently recommended first line therapeutics—systemic chemotherapeutics: alkylating agents (cisplatin or carboplatin) and M-phase specific tubulin inhibitors (paclitaxel or docetaxel), which have serious iatrogenic consequences for all proliferating cells including those of the immune system; hence delivering extremely toxic compounds to the patients. AVEC could facilitate significant reduction in doses of these first line systemic chemotherapeutics, or replace them as first line in cases of inoperable or cisplatin resistant, clinically advanced stages of ovarian cancer. This would dramatically benefit our patients.

The above considerations are critical for the correct qualification of the patient for designing and attaining high efficacy of this novel therapy—RAAVIIT.

## Abbreviations

OEC: ovarian epithelial cancer
HOSE: human ovary surface epithelial cells
HAE: human artery endothelial cells
HER-2: human epidermal growth factor receptor 2
HBsAg: hepatitis B virus surface antigen
HBV: hepatitis B virus
VLP: virus like particle
CTL: cytotoxic lymphocyte
NKC: natural killer cell
NMR: nuclear magnetic resonance
XRFS: x-ray reflection fluorescence spectroscopy
FCM: fluorescent flow cytometry
FACS: fluorescent activated cell sorting
MFM: magnetic flow cytometry
MACS: magnetic activated cell sorting
RAAVIIT: redirected, accelerated, amplified, vaccination-induced, immunotherapy
AVEC: antibody vaccine engineered construct
